# The change of HCN1/HCN2 mRNA expression in peripheral nerve after chronic constriction injury induced neuropathy followed by pulsed electromagnetic field therapy

**DOI:** 10.18632/oncotarget.13584

**Published:** 2016-11-25

**Authors:** Hui Liu, Jun Zhou, Lianbing Gu, Yunxia Zuo

**Affiliations:** ^1^ Department of Anesthesiology, Jiangsu Cancer Hospital, Jiangsu 210009, China; ^2^ Department of Anesthesiology, West China Hospital, Sichuan University, Sichuan 610041, China; ^3^ Department of Rehabilitation, West China Hospital, Sichuan University, Sichuan 610041, China

**Keywords:** hyperpolarization-activated cyclic nucleotide-gated cation channels (HCN), chronic constriction injury (CCI), pulsed electromagnetic field (PEMF)

## Abstract

Neuropathic pain is usually defined as a chronic pain state caused by peripheral or central nerve injury as a result of acute damage or systemic diseases. It remains a difficult disease to treat. Recent studies showed that the frequency of action potentials in nociceptive afferents is affected by the activity of hyperpolarization-activated cyclic nucleotide-gated cation channels (HCN) family. In the current study, we used a neuropathy rat model induced by chronic constriction injury (CCI) of sciatic nerve to evaluate the change of expression of HCN1/HCN2 mRNA in peripheral nerve and spinal cord. Rats were subjected to CCI with or without pulsed electromagnetic field (PEMF) therapy. It was found that CCI induced neural cell degeneration while PEMF promoted nerve regeneration as documented by Nissl staining. CCI shortened the hind paw withdrawal latency (PWL) and hind paw withdrawal threshold (PWT) and PEMF prolonged the PWL and PWT. In addition, CCI lowers the expression of HCN1 and HCN2 mRNA and PEMF cannot restore the expression of HCN1 and HCN2 mRNA. Our results indicated that PEMF can promote nerve regeneration and could be used for the treatment of neuropathic pain.

## INTRODUCTION

Neuropathic pain is usually defined as a chronic pain state caused by peripheral or central nerve injury as a result of acute damage or systemic diseases. It is characterized by unpleasant abnormal sensation (dysesthesia), increased response to painful stimuli (hyperalgesia) and pain in response to a stimulus that does not normally provoke pain (allodynia) [1]. Recent studies showed that the frequency of action potential in nociceptive afferents is affected by the activity of hyperpolarization-activated cyclic nucleotide-gated cation channels (HCN) family [2]. HCN represents the molecular correlation of Ih current, a cation current activated by membrane hyperpolarization contributing to the formation of resting membrane potential. All four HCN members (HCN1, HCN2, HCN3 and HCN4) have been found in central and peripheral nervous system, where they are associated with synaptic integration, neuronal excitability and the formation of resting membrane potentials [3]. HCN1 and HCN2 are the most expressed isoforms in primary somatosensory neurons [4; 5].

Neuropathic pain remains difficult to treat. Conventional analgesics such as non-steroidal anti-inflammatory drugs and opioids are often not effective in reducing neuropathic pain. Due to the lack of effective therapeutic agents, it is necessary to search for potential alternative therapies for this kind of diseases. Recent studies have shown that electrical or magnetic application may have positive impact on the regeneration of injured peripheral nerves [6]. However, it is not clear whether pulsed electromagnetic field (PEMF) could relieve neuropathic pain. Because PEMF induces small electrical currents that may depolarize, repolarize and hyperpolarize neurons [7, 8]. We hypothesized that the energy released by PEMF might potentially affect the expression of HCN channels.

Chronic constriction injury (CCI) of sciatic nerve induced painful neuropathy is a commonly accepted model for induction of neuropathic pain in experimental animals [9]. In the current study, we investigate the expression of HCN1/HCN2 mRNA in peripheral nerve system and spinal cord in a CCI induced neuropathic pain rat model. We explore whether PEMF have a therapeutic effect in CCI induced neuropathy and whether there is a change in the expression of HCN1/HCN2 after PEMF therapy. Our study provides a rational for using PEMF in the treatment of neuropathic pain.

## RESULTS

### CCI shortened the PWL and PWT, while PEMF prolonged the PWL and PWT

**Figure 1:**
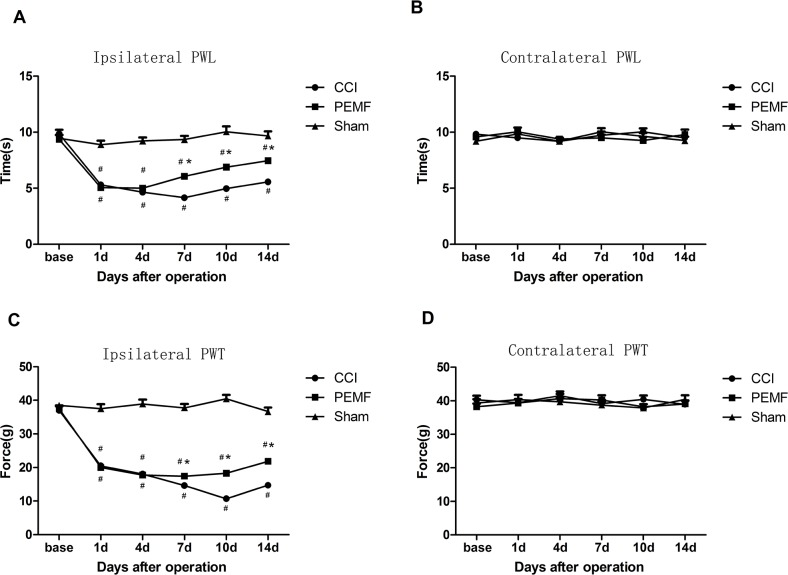
**A.** Ipsilateral paw withdrawal latency (PWL) to thermal stimulation: The time of PWL in group PEMF and group CCI were significantly shorter than PWL in group Sham after surgery; the time of PWL in CCI group was shorter than that in PEMF group on day 7,day 10 and day 14 after surgery. **B.** Contralateral paw withdrawal latency (PWL) to thermal stimulation: There were no differences among three groups. **C.** Ipsilateral paw withdrawal threshold (PWT) to mechanical stimulation: PWT in PEMF group and CCI group were significantly lower than PWL in Sham group after surgery; PWL in PEMF group was higher than that in CCI group on day 7,day 10 and day 14 after surgery. **D.** contralateral PWT to mechanical stimulation: There were no differences among three groups. *p<0.01,PEMF group vs CCI group; ^#^ p<0.05vs Sham group.

We have tested the hind paw withdrawal latency (PWL) to thermal stimulation and the hind paw withdrawal threshold (PWT) to mechanical stimulation. In the hind limb where surgery was performed, the time of PWL in rats with sham CCI and sham PEMF was around 10 seconds. The PWL shorten in rats underwent CCI with or without PEMF, especially in the first few days and recovered from the 7^th^ day of surgery. The PWL in PEMF group and CCI group was shorter than that of Sham group on the 7d,10d and 14d after injury (P<0.01). PWL of PEMF group were longer than that of CCI group from 7d to 14d (P<0.01) (Figure 1A). In the contralateral limb where there was no surgery was performed, there was no change in the PWL observed (Figure 1B). These results indicated that CCI shorten the PWL and PEMF can promote the recovery of injury and prolong the PWL to thermal stimulation.

Similar changes were observed in PWT. PWT decreased after CCI induced neuropathy. As shown in Figure 1C and 1D, PWT of PEMF group and CCI group were significantly lower than that of Sham group (P<0.01). PEMF treatment increased the PWT of left hind paw after sciatic nerve injury from 7d to 14d compared with that of CCI group (P<0.01).

### CCI induced nerve degeneration and PEMF promoted nerve regeneration

**Figure 2:**
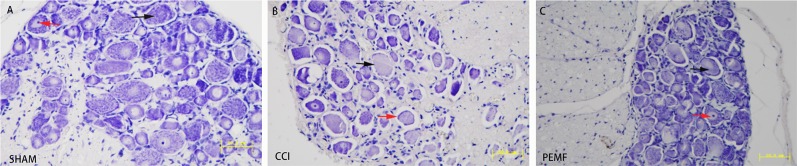
Nissl staining of the dorsal root ganglion **A.** SHAM group. Normal dorsal root ganglion on Nissl staining. Nissl body in the cytoplasm of nerve cells (black arrow). Normal nucleus in nerve cells (red arrow). **B.** CCI group. Most of the Nissl bodies lost in the nerve cells (black arrow). Lost of nucleus was seen in most of nerve cells (red arrow). **C.** PEMF group. Almost normal dorsal root ganglion. Nissl body in the cytoplasm of nerve cells (black arrow). Normal nucleus in nerve cells (red arrow).

To examine histological change after CCI with and without PEMF, Nissl staining was performed on dorsal root ganglions. It was found that the dorsal root ganglions have normal histology in SHAM group, showing abundant Nissl bodies in the cytoplasm of nerve cells and normal nucleoli (Figure 2A). However, in the CCI dorsal root ganglions, degenerated nerve cells was found as indicated by the disappearance of Nissl bodies and lost of nucleoli 14 days after CCI (Figure 2B). The recovery of nerve cell morphology was observed in PEMF treated group. We observed the re-appearance of Nissl bodies and nucleoli (Figure 2C).

### CCI lowers the expression of HCN1 and HCN2 mRNA

**Figure 3:**
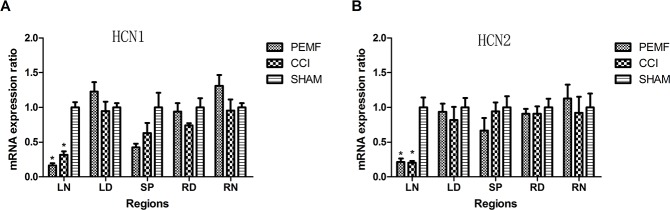
**A.** HCN1 mRNA expression in different regions of nerves 14 days after surgery in three groups. HCN1 mRNA expressions of left sciatic nerve in group PEMF and group CCI were significantly lower than that in group SHAM. **B.** HCN2 mRNA expression of different areas 14 days after operation in three groups. HCN2 mRNA expressions of left sciatic nerve in group PEMF and group CCI were lower significantly than that in group SHAM. *p<0.01;^#^ p<0.05. Note: LN (left sciatic nerve), LD (left dorsal root ganglion), SP (spinal cord), RD(right dorsal root ganglion), RN(right sciatic nerve).

**Figure 4:**
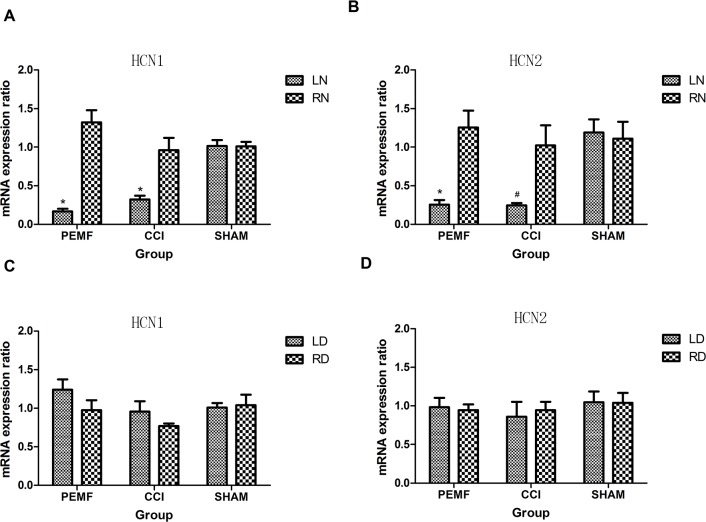
**A.** HCN1 mRNA expressions of left and right sciatic nerve 14 days after operation in three groups. HCN1 mRNA expressions of left sciatic nerve in group PEMF and group CCI decreased significantly compared with that of right sciatic nerve. **B.** HCN2 mRNA expressions of left sciatic nerve in group PEMF and group CCI decreased significantly compared with that of right sciatic nerve. **C.** HCN1 mRNA expressions of left and right dorsal root ganglions 14 days after operation in three groups. **D.** HCN2 mRNA expressions of left and right dorsal root ganglions 14 days after operation in three groups. *p<0.01; ^#^ p<0.05.

The expression of HCN1/HCN2 mRNA was examined after surgery. In the left sciatic nerve where neuropathy was induced by CCI, the expression of HCN1/HCN2 mRNA decreased. In other regions (LD: left dorsal root ganglion, SP: spinal cord, RD: right dorsal root ganglion, RN: right sciatic nerve) of nerves where no neuropathy was induced, no change of HCN1/HCN2 mRNA was observed. As shown in Figure 3, the decreased expression of HCN1/HCN2 mRNA has statistical significance. Comparing the change of HCN1/HCN2 mRNA expression in PEMF, CCI and SHAM groups, it was found that in group PEMF, HCN1 and HCN2 of left sciatic nerve was lower than that of right sciatic (P<0.001 for HCN1, P=0.002 for HCN2); in group CCI, HCN1 and HCN2 of left sciatic nerve was lower than that of right sciatic (P=0.005 for HCN1, P=0.038 for HCN2) (Figure 4A and 4B). There was no change of expression of HCN1/HCN2 mRNA in the left and right dorsal root ganglion (Figure 4C and 4D).

## DISCUSSION

Our study revealed the change of expression of HCN1/HCN2 mRNA in the left/right sciatic nerve, left/right dorsal root ganglion (DRG) and spinal cord after CCI induced neuropathy. Fourteen days after CCI, the HCN1/HCN2 mRNA in the injured left sciatic nerve significantly decreased compared with that in the right uninjured sciatic nerve. However, there were no changes in other regions such as DRG and spinal cord. This result was not consistent with previous reports with spinal nerve ligation (SNL) induced neuropathic pain model, where HCN1 and HCN2 mRNA and protein decreased in DRG [11; 12], HCN1 mRNA expression declined to the least level at the 14th day and recovered at day 28 after spinal nerve was ligated. It was also reported that HCN2 mRNA and protein also diminished in DRG after spinal nerve ligation [13], although we didn't find any changes of HCN1/HCN2 mRNA expressions in DRG in our current study. We speculate that the decreased HCN1 and HCN2 mRNA in left sciatic nerve was due to the injury of some part of fibers of sciatic nerve. Since the sciatic nerve is far away from DRG, the injury cannot cause HCN1/HCN2 changes in DRG.

In CCI induced neuropathy, the injured sciatic nerve leads to increased spontaneous firing or alterations in the conduction of neurotransmitter, resulting in chronic pain or persistent pain [14]. Discharges from injured peripheral nerve also plays a crucial role in the initiation and maintenance of neuropathic pain, although central nervous system (CNS) are responsible for hypersensitivity and miscoding of sensory information [15]. It was well known that thermal and mechanical pain is mediated by different types of sensory neurons [16; 17]. Small peripheral nerve fibers regulate the sensations of thermal pain, which is triggered by input from peripheral nociceptors [18]. Some electrophysiological and molecular evidences suggest that HCN1-based channels significantly contribute to hyperpolarization-activated cyclic nucleotide gated currents Ih, supporting abnormal spontaneous firing after nerve injury, particularly in large diameter mechanosensitive fibers [19]. HCN2 expression in nociceptive neurons plays a key role in adjusting the generation of action potentials in reaction to inflammation, and HCN2 exerts fundamental effects in managing the excitability of nociceptor at the cellular level [2].

Our study demonstrated that PEMF has effects on relieving neuropathic pain in CCI induced neuropathy rat model. It promotes PWL to thermal stimulus and PWT to mechanical stimulus, which means that PEMF can decrease the hypersensitivity induced by neuropathy. PMF could maintain the electrophysiological characteristics and morphological properties of the injured sciatic nerve in rat [20]. Static and time varying magnetic field can influence the pain perception of animal and human behaviors [21]. PEMF with magnetic field at the intensity of 200μT could increase the latency to thermal stimulation in land snails [22] and mice [23]. Magnetic field has been applied to improve neuronal recovery after injury, to lessen neuropathic pain [24], due to the stimulation of the central and peripheral neural systems. Pulsed magnetic field (PMF) has been used to treat the peripheral nerve injury for years, for it can accelerate the functional recovery of injured peripheral nerve [25]. PMF can ameliorate the hyperpolarization of after potentials and delayed depolarization of nerves [26]. PMF can also promote peripheral nerve regeneration in different experimental animal models [27; 28], because axons of neuron are sensitive to electrical and magnetic stimulation [24]. However, PEMF had no effect on the change of expression of HCN1 and HCN2 mRNA after CCI. It is most likely that the recovery of expression of HCN1/2 mRNA needs longer time and could not be detected in the current experiments. This warrants further investigation. The change of HCN1/2 protein expression and its relation to mRNA expression is also worth exploring.

Taken together, our results indicated that nerve degeneration caused by CCI could be restored by PEMF. This study provided a rational for using PEMF for the treatment of neuropathic pain.

## MATERIALS AND METHODS

### Establishing a CCI induced neuropathy rat model

This study was approved by the Ethical Committee of HuaXi Medical Center of SiChuan University. Male Sprague-Dawley rats, 300-350g, were purchased fromthe Animal Center of SiChuan Province in China. Rats were raised and cared by the specific personnel in the Animal Experiment Center of HuaXi Medical Center in SiChuan University in rooms with control temperature and 12 hour light/dark cycle. They were given free access to food and water.

Rats were given chronic constriction injury (CCI) surgery or sham operation on left sciatic nerve. In CCI animal model, ligation of left sciatic nerve was performed as described by Bennett and Xie [9]. Briefly, Rats were anesthetized with sodium pentobarbital (30mg/kg, i.p.). The left sciatic nerve at the level of mid- thigh was exposed. Four loose ligatures (4.0 chromic gut) were performed around the sciatic nerve with 1mm spacing. The rats were randomly divided into three groups with 11 rats in each group: 1) SHAM Group: Rats were given sham operation and received sham PEMF. 2) CCI Group: Rats received CCI surgery and sham PEMF. 3) PEMF Group: Rats were given CCI surgery followed by PEMF therapy. PEMF therapy was applied on the whole body of rat. The rat was put in a plastic cage (30*20*18 cm) covered with hollow frames, then the cage was put into the therapeutic area of the pulsed electromagnetic therapy machine (Tianjin medical instrument factory, China). The parameters for PEMF were set as 3.8mT, 8Hz, 30min. The treatment began from the first day and sustained 14 days after surgery.

### Hind paw withdrawal latency (PWL) test

The hind paw withdrawal latency (PWL) to thermal stimulation was examined using a Plantar apparatus (Plantar Test-7370, UGO BASILE, Italy).

The rats were placed in an organic glass cage with three separated small rooms upon the floor of glass. A radiant heat source was aimed at the plantar of hind paw, and the latency from starting the stimulus to the time of withdrawing of the hind paw was recorded (the maximal latency was set as 30 seconds).

### Hind paw withdrawal threshold (PWT) test

The hind paw withdrawal threshold (PWT) to mechanical stimulation was measured by a Plantar apparatus (Plantar Von Frey-37400, UGO BASILE, Italy). During the test, the rats were put in an organic glass cage with two small separated rooms with the floor of metal mesh. A probe with a blunt tip was aimed at the plantar of hind paw, and the intensity of pressure (the maximal was 50 grams) was increasing continuously until the rat removing its hind paw. The machine automatically recorded the number of pressure.

We repeated PWL and PWT tests three times with 5 minutes intervals for each side consecutively and the three measurements per side were averaged as the data of PWL and PWT.

### RNA extraction and real time PCR procedures

Fourteen days after CCI, rats were euthanized and dorsal root ganglions (DRGs) from left lumber 4-6 (L4-6)) and right lumber 4-6 (R4-6) nerves, the exposed and ligated sciatic nerves and spinal cord segment lumber 4-6 were collected. All tissues were homogenized in a ceramic grinding bowl and total RNA was extracted using TRIzol (Invitrogen) according to the manufacturer's instructions.

Real time PCR was performed in an FTC2000 Sequence Detector (FUNFLYN, CA). Nucleotide sequences for primers and probes were as follow:

1) HCN1: GENEBANK ID LOCUS: NM_053375
  HCN1-F: CTCTTACTTTGGAGAAATATGC
  HCN1-R: TAGAGTTTTTCTTGCCTATCCG
  Probe: CTTGGAGGAATATCCAATGATG
2) HCN2: LOCUS: NM_053684
  HCN2-F: CTACAGCGACTTCAGGTTCTAC
  HCN2-R: TAACAATGCCGGTGCGAAAGTT
  Probe: CGTCTTCAACGTGGTCTCGGAC
3) ACTB: LOCUS NM_031144
  ACTB-F: AGATGACCCAGATCATGTTTGA
  ACTB-R: GAGTCCATCACAATGCCAGTGG
  Probe: CCATCCAGGCTGTGTTGTCCCT

HCN1/HCN2 cDNA derived from the rat total RNA samples was used for the real-time PCR analysis. A 30-μl volume of PCR reaction mixture contained 5μl of cDNA, 1μl of forward primer, 1μl of reverse primer, 1μl of probe, 0.3μl of Taq enzyme (5u/μl), 0.36μl dNTP(25mM), 3μl MgCl_2_(25mM),3μl buffer and 15.34 μl ddH_2_O. The thermocycling conditions in the amplification were 2 min at 94°C, and 45 cycles of 20 sec at 94°C, 30 sec at 55°C and 40 sec at 60°C. The melting curves were detected and the standard curve was acquired based on the quantity of cloned HCN DNA. Relative expressions of HCN1 and HCN2 mRNA were calculated using the method of comparative threshold cycle [10].

### Nissl staining

After the animals were sacrificed, and dorsal root ganglions (DRGs) were removed and immersed in 4% formaldehyde overnight. The DRGs were embedded in paraffin and cut into 4 μm sections. DRGs sections were washed by distilled water, and stained with 0.5% thionine for ten minutes at 50°C, differentiated in 95% ethyl alcohol for fifteen minutes. The sections were dehydrated in 100% alcohol for five minutes twice, then cleared in xylene for five minutes twice. Finally, all the DRG sections were mounted by resinous medium.

### Statistics

Data were expressed as means ± SE (Standard error). PWL and PWT were analyzed with repeated measures of General linear model and one-way ANOVA. HCN1 and HCN2 mRNA expression were analyzed with one-way ANOVA or T-test. Differences were considered significantly at P*<*0.05.
